# *In-vitro* anti-acne activity of *Teucrium oliverianum* methanolic extract against *Cutibacterium acnes*

**DOI:** 10.3389/fphar.2024.1388625

**Published:** 2024-10-03

**Authors:** Abdullah A. Al-Ghanayem

**Affiliations:** Department of Clinical Laboratory Science, College of Applied Medical Sciences, Shaqra University, Shaqra, Saudi Arabia

**Keywords:** antibacterial, IL-1β, INF-γ, LC-MS, reactive oxygen species, TNF-α

## Abstract

**Background:**

Acne vulgaris is a skin infection widely seen in adolescents between 10–19 years with males affected more than females. It mainly affects the face but may also affect the back and chest. The symptoms vary with mild acne manifesting as comedones and moderate acne as inflammatory lesions (papulopustular), nodules, and mild scarring while severe acne has the same symptoms that have not subsided within 6 months of treatment. Various treatments including topical medications containing different antibiotics are used to treat acne. Recently, herbal treatments have been shown as better alternatives to conventional treatment. *Teucrium oliverianum* Ging. ex Benth (*Lamiaceae*) is traditionally used for skin infections such as wound healing and biofilm formation.

**Methodology:**

Methanolic extract of *T. oliverianum* was subjected to liquid chromatography-mass spectrometry (LC-MS) analysis, and its antibacterial effect against *Cutibacterium acnes*. The anti-acne, anti-inflammatory, and antioxidant effects were also assessed using HaCaT cells infected with *C. acnes.* The cytotoxicity of the extract was evaluated using a neutral red uptake assay, and anti-inflammatory effects were determined by measuring TNF-α, IL-1β, INF-γ, and COX2 inhibition. The antioxidant action was assessed by ROS generation in HaCaT cells infected with *C. acnes*.

**Results:**

LC-MS analysis of the extract showed the presence of 16 different metabolites with L-carnitine, esculin sesquihydrate, and gamma-linoleic acid as major metabolites. The methanolic extract of *T. oliverianum* showed an antibacterial effect against *C. acnes* with an IC_50_ value of 263.2 μg/mL. The extract attenuated the cytotoxicity of *C. acnes* on the HaCaT cell and the IC_50_ was found to be 676.2 μg/mL. It also decreased dose-dependently the expression of TNF-α, IL-1β, INF-γ, and inhibited COX2 in the HaCaT cells infected with *C. acnes*. It also decreased the generation of reactive oxygen species.

**Conclusion:**

The results support the use of *T. oliverianum* as an anti-acne agent but it possesses mild antibacterial action. It showed anti-inflammatory effects in HaCaT cells infected with *C. acnes*. It is also an effective antioxidant and decreased the generation of reactive oxygen species. Comparison of the anti-acne effects and adverse reactions of extract with other treatments will provide more insight into its clinical efficacy and toxicity.

## 1 Introduction

Acne vulgaris is a major skin infection that affects the adolescent population affecting males more than females. Adolescent acne is seen between 10–19 years and peaks at 17 years of age. Acne also affects adults (adult acne) and the number of affected individuals aged 25 years or more is increasing. Acne causes psychological distress, mood swings, depression, and violent behavior and affects the quality of life (Purdy and Berker, 2011). The clinical manifestations include comedones, papules, nodules, pustules, and cysts due to high sebum secretion, hyperkeratinization, hormonal changes, and bacterial infection (Heath and Usatine, 2021). The causative agent for acne vulgaris is *Cutibacterium acnes*, which is an important member of the skin microbiota. It acts by stimulating the immune system to produce different enzymes and molecules that increases the seborrhea, and hyperkeratinization in the pilosebaceous unit that results in inflammation (Boyanova, 2023). In individuals suffering from acne, *C. acnes* accounts for about 30% of the face microbiota (Dréno et al., 2020). *C. acnes* are capable of forming biofilms during infection that penetrate into sebum and form micro-comedones due to increased cohesiveness of corneocytes (Hazarika, 2021). Antibiotics such as tetracycline, erythromycin, and clindamycin-based topical drugs and related antimicrobial agents are used to control acne-related infections. These treatment options have side effects and may lead to the development of antibiotic resistance (González-Mondragón et al., 2022). Long-term, low-concentration use of antibiotics increases resistance. One reported mutation is Macrolide-resistant *C. acnes* with 23S rRNA mutation (Nakase et al., 2016). Furthermore, antibiotics have poor efficacy against *C. acnes*. Hence, the development of drugs with fewer side effects and multiple mechanisms of action such as anti-inflammatory, antioxidant, and additional antimicrobial effects would be beneficial in acne treatment. To overcome antibiotic resistance and adverse side effects, essential oils and phytochemicals have been studied as alternative antimicrobial agents.

*Teucrium* (Family- Lamiaceae), locally called “Qassapa” in Arabic, is an ornamental perennial plant commonly known as Germander in English. It is rich in essential oils and widely used in traditional medicine. *Teucrium* species has been reported for antioxidant, analgesic, antidiabetic, anti-inflammatory, antimicrobial, antiulcer, anti-cancer, antispasmodic, insecticidal, and anthelmintic properties (Fatima, 2016). These species are rich in sterols, tannins, flavonoids, Iridoids, coumarins, saponins, and cardiac glycosides and these metabolites are reported for the mentioned above-mentioned effects (Arzi et al., 2011). *Teucrium polium* L. has been reported to treat skin disorders (Alizadeh et al., 2011). In Saudi Arabia, this plant is applied locally as a paste to treat acne. In the present study, *Teucrium oliverianum* Ging. ex Benth was extracted using methanol, and the phytochemicals were analyzed using liquid chromatography-mass spectrometry. The extract was studied for antiacne activity and also evaluated for cytotoxicity on human keratinocytes. Further, cyclooxygenase-2 (COX-2) activity, expression of cytokines – tumor necrosis factor-α (TNF-α), interleukin-1β (IL-1β), and interferon-γ (INF-γ) expression, and antioxidant activities were determined.

## 2 Materials and methods

### 2.1 Chemicals and bacterial culture

Analytical-grade methanol (SD fine chemicals, India) was used for the extraction of plant material. Acetonitrile and ammonium formate (HPLC grade) procured from Merck was employed for the LC-MS analysis of the extract. The media used for the evaluation of antimicrobial activity were purchased from HiMedia (India). The diluting solvent for the extract was dimethyl sulfoxide (DMSO) and it was from HiMedia (India). The bacterial culture *C. acnes* (MTCC 1951) was used to determine antibacterial effects and to infect the human keratinocyte (HaCaT) cells.

### 2.2 Extraction of the plant material

Aerial parts of *T. oliverianum* Ging. ex Benth that included leaves, flowers, and stems were collected in June 2023 locally (25.2476° N, 45.2525° E), and a voucher specimen (No. SU/CAMS-MDL/09/2023) was preserved in the departmental herbarium for reference. The dried aerial parts (5 g) were extracted using a solvent mixture (50 mL) of 80% methanol in distilled water using a rocker shaker (VRN-380A, Gemmy, Taiwan) for 24 h. The mixture was filtered under using Whatman filter paper no. 2 without any vacuum followed by centrifugation and dried in an oven (Thermo Fisher Scientific, United States) at 40°C. The extract was collected in a microcentrifuge tube and kept at 4°C until use.

### 2.3 Liquid chromatography-mass spectrometry (LC-MS) analysis

The LC-MS analysis was carried out using Waters LC instrument (Waters Alliance e2695/HPLC-TQD Mass Spectrometer) with a SUNFIRE C18 column using acetonitrile (Merck, India) and ammonium formate buffer (Merck, India) as solvents at a 0.2 mL/min flow rate and detection at 280 nm using a photomultiplier detector. The extract was centrifuged at 12,000 rpm for 10 min before injection. A volume of 20 µL was injected manually into the instrument. The gradient mode started with 5% acetonitrile and increased to 80% with a corresponding concentrations of ammonium formate. Ionization modes between m/z 150 and 2,000 were used to record the spectra, and the compounds were identified using ReSpect for phytochemicals (http://spectra.psc.riken.jp/).

### 2.4 Antibacterial activity

The Inhibitory Concentration 50% (IC_50_) was determined using 0.5 McFarland standard dilutions of *C. acnes*– MTCC 1951 culture. The optical density of culture was adjusted to 0.5 McFarland’s at 600 nm. From the isolated colonies on brain heart infusion agar (Himedia, India), *C. acnes* was inoculated in brain heart infusion broth (Himedia, India) and incubated at 37°C for 72 h in an anaerobic jar (Oxoid, Thermo Fischer Scientific, MA, United States) with a GasPaK™. Diluted log culture (100 µL) was taken into a microcentrifuge tube (1 mL), with prepared extract at different concentrations in geometrical dilutions (15.12 to 500 μg/mL), followed by incubation for 24 h in anaerobic jar with a GasPaK™. The extract was diluted in 10% dimethyl sulfoxide (DMSO) and a blank containing only 10% DMSO without the extract was used for comparison (100% cell viability). After incubation, the contents were transferred to the 96 well plates, and turbidity was recorded using an ELISA Plate Reader (iMark Biorad) at 630 nm. Ciprofloxacin at a single concentration of 10 μg/mL (Rapidlife, India) was used as a positive control (Dell’Annunziata et al., 2022).

### 2.5 The effect of *Cutibacterium acnes* on HaCaT cell viability

The cells (HaCaT) were cultured as mentioned above. Then, 5 µL of the live *C. acnes* cultured (0.5 McFarland standard) was added to the defined well of the groups. The volume of the media was 100 µL. The extract at various dilutions (50, 100, and 200 μg/mL) was added and incubated for 24 h, and an NRU assay was performed (Vichai and Kirtikara, 2006). Solvent (10% DMSO) without the extract served as control.

### 2.6 Cytotoxicity assay of *Teucrium oliverianum* extract on HaCaT cells by neutral red uptake (NRU) assay

Cytotoxicity was determined on human epidermal keratinocyte (HaCaT) cells (NCCS, Pune, India) (Rodrigues et al., 2023) at 75% confluency. The cells were grown in a T-75 flask with DMEM (Dulbecco’s Modified Eagle Medium-AT149-1L) supplemented with 10% FBS (Fetal Bovine Serum - HIMEDIA-RM 10432) and 1% antibiotic solution at 37°C with 5% CO_2_ to obtain 75% confluency. The cell layer was rinsed with phosphate buffer and trypsinized with 0.25% trypsin in 0.2 g/L EDTA till the cells were detached. The DMEM with 10% FBS was added to flush out the cells followed by centrifugation at 900 rpm for 5 min. The cells were suspended in DMEM and the cell number was adjusted. The cells (5,000–8,000 cells/well) were cultured in 96 well plates for 24 h in DMEM medium supplemented with 10% FBS and 1% antibiotic solution at 37°C with 5% CO_2_. Next day, medium was removed and fresh culture medium was added to each well of the plate. The extract (5 µL) at different concentrations (1 to 1,000 μg/mL) in 10% dimethyl sulfoxide (DMSO) (Loba Chemie, India) was added to the cells and incubated for 24 h. Neutral red (SRL Chem-36248) (40 μg/mL in phosphate buffered saline – 100 µL) was added to the defined wells and incubated in a CO_2_ incubator (Heal Force-Smartcell CO_2_ Incubator-Hf-90) for 1 h. The medium was discarded, and neutral red was dissolved in 100 µL of NRU destain solution. Control was DMSO without the extract. Finally, plates were read at 550/660 nm using Elisa Plate Reader (iMarkBioRad-CA, United States), and IC_50_ values were calculated. Each experiment was carried out in duplicates and the experiments were repeated four times (n = 4).

### 2.7 COX-2 enzyme inhibition assay

Sample dilutions of 0.2 to 200 μg/mL in Buffer (TrisCl buffer, 100 mM, pH 8.0) were prepared. Reaction buffer (Enzyme in Tris/heme/phenol; 100 mM/1 μM/1 μM buffer) was placed in a defined well of a 96-well plate. The reaction was initiated by adding 5 µL substrate (Arachidonic acid, 10 mM) and 5 μL of N,N, N′, N′-tetramethyl-p-phenylenediamine (Loba-Chemie, India) solution (17 mM) and the plate was incubated at room temperature for 10 min. The absorbance was recorded at 595 nm (iMark, BioRad). Celecoxib (25 µM) was used as a standard COX-2 inhibitor while blank was 10% DMSO without the extract as control (Kang et al., 2007).

### 2.8 TNF- α expression analysis

The cell culture was done as mentioned above and cells were treated with extract. After incubation, cells were harvested by centrifugation, the cell pellet was subjected to sonification using phosphate buffer saline followed by centrifugation. The supernatant was taken and the experiment was performed using a commercial kit following the manufacturer’s instructions (GENLISA™ Human TNF α ELISA- Cat No: KB1145). Standard (TNF α- 1 μg/mL) and supernatant from cells treated with three concentrations (50, 100, and 200 μg/mL) were taken in the wells, sealed, and incubated for 2 h at room temperature. Solvent (10% DMSO) served as control. The plate was then washed using a wash buffer (1X). A diluted detection antibody (Biotin Conjugated Detection Antibody) was added, sealed, and incubated for an hour at room temperature. The plate was rewashed using the same buffer, followed by the addition of diluted streptavidin-HRP -HRP solution and incubated for an hour. The plate was rewashed using the buffer, and 3,3′,5,5′-Tetramethylbenzidine substrate was added and incubated for 30 min in the dark. The reaction was terminated using a stop solution, and absorbance was recorded at 450 nm.

### 2.9 IL-1β expression analysis

The experiment was performed using a commercial kit following the manufacturer’s instructions (GENLISA™ Human IL-1β ELISA- Cat No: KB1063). Standard (IL-1β - 1 μg/mL) and supernatant from the extract treated cells were added to the wells, followed by incubation for 2 h at room temperature. The same procedure mentioned above under TNF-α TNF-expression was used with DMSO (10%) as control.

### 2.10 INF-γ expression analysis

This was done using a commercially available kit (IFN-γ- GENLISA TM Human IFN- γ ELISA-KB1053). Standard (IFN- γ – 0.2 μg/mL) and supernatant from extract treatment as mentioned above were added to the plate, sealed, and incubated for 2 h at room temperature. The same procedure mentioned above under TNF-α expression was used.

### 2.11 Measurement of reactive oxygen species (ROS) generation by flow cytometry

The HaCaT cells were plated in 6 well plates at a density of 50,000–100,000 cells/well in 1 mL DMEM medium supplemented with 10% FBS and 1% Antibiotic solution, and incubated for 24 h at 37°C & 5% CO_2_. After incubation, old medium was removed and the fresh culture medium was added before treatment. Cells were treated with extract at different concentrations (50–200 μg/mL) and *C. acnes* (0.5% of McFarland’s standard) followed by incubation for 24 h. The cells were then harvested with trypsin EDTA, washed using PBS, and dispensed in 100 μL PBS with 2 μM DCFDA (dichlorodihydrofluorescein diacetate). The samples were acquired in a Flow Cytometer (BD FACS Calibur, United States) within an hour. DMSO (10%) without the extract was taken as control. The results were analyzed by using Flowing Software version 2.5.1.

### 2.12 Statistical analysis

Data are expressed as mean ± SEM. ANOVA followed by Bonferoni’s test was used to analyze the data using InStat software.

## 3 Results

### 3.1 Extraction and phytochemical analysis

**FIGURE 1:**
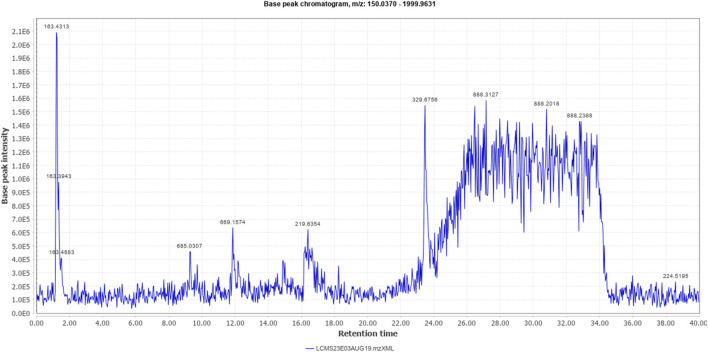
Total ion chromatogram (positive mode) of *Teucrium oliverianum* extract.

**FIGURE 2:**
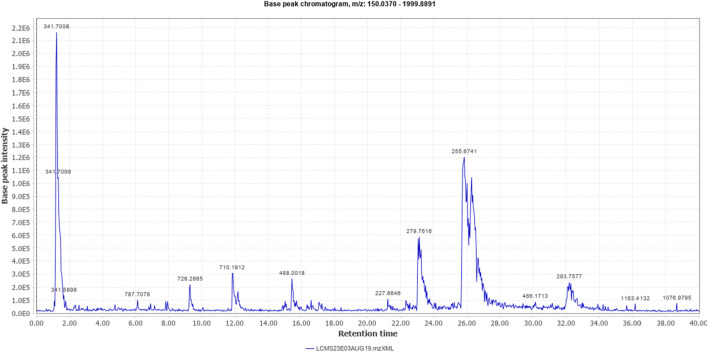
Total ion chromatogram (negative mode) of *T. oliverianum* extract.

The % yield of the extract was 9.18% w/w. The LC-MS analysis helped identify various metabolites in *T. oliverianum* methanolic extract (Figures 1, 2). A total of 16 metabolites, 5 in the positive and 11 in the negative modes, were revealed (Tables 1, 2). Some of the important metabolites identified were kaempferol, nicotine, luteolin, and linoleic acid.

**TABLE 1:** Predicted phytoconstituents in positive mode.

R. Time	Score	Compound name	Formula	Exact mass	Observed mass	Mass diff	Chemical structure
1.35	0.797	L-Carnitine	C_7_H_16_NO_3_	162.113	163.394	1.2813	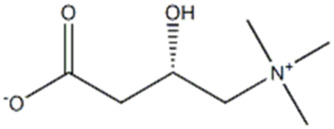
1.52	0.639	Hinokitiol	C_10_H_12_O_2_	164.083	163.468	0.6147	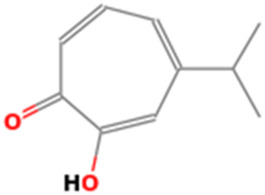
9.27	0.411	3′-dephosphocoenzyme A	C_21_H_35_N_7_O_13_P_2_S	687.148	685.030	2.1173	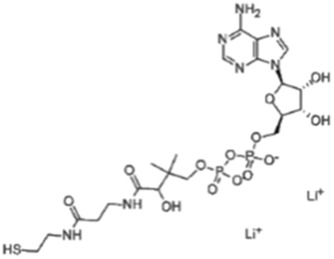
23.4	0.594	Adenosine-3′,5′-cyclicmonophosphate	C_10_H_12_N_5_O_6_P	329.21	329.675	0.4656	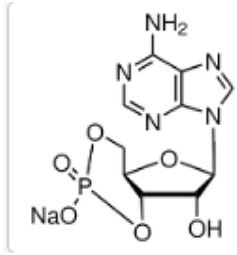
26.4	0.718	Safranine	C_20_H_19_N_4_	315.16	313.728	1.4317	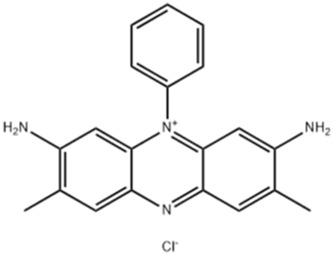
34.0	0.505	Cyanidin-3-O-(6″-O-(E-P-coum)-2″-O-(beta-xylopyranosyl)-beta-glucopyranoside)-5-O-beta-glucopyranoside trifluoroacetate salt	C_41_H_45_O_22_	889.24	888.275	0.9642	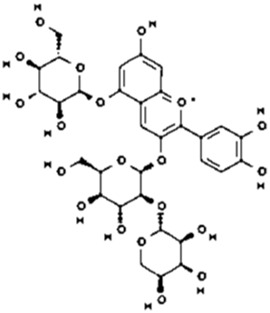

**TABLE 2:** Predicted phytoconstituents in negative mode.

R. Time	Score	Compound name	Formula	Exact mass	Observed mass	Mass diff	Chemical structure
1.23	0.941	Galactinol dihydrate	C_12_H_22_O_11_	342.116	341.7008	0.4152	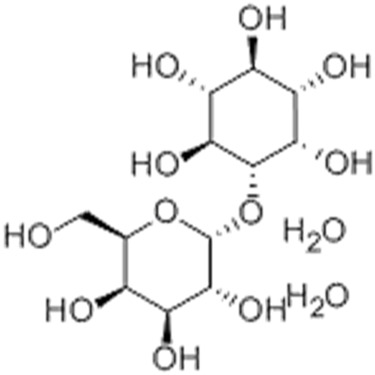
1.33	0.915	Esculin sesquihydrate	C_15_H_16_O_9_	340.079	341.7008	1.6218	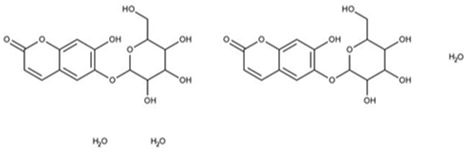
7.82	0.593	Kaempferol-3-glucuronide	C_21_H_18_O_12_	462.079	461.6202	0.4588	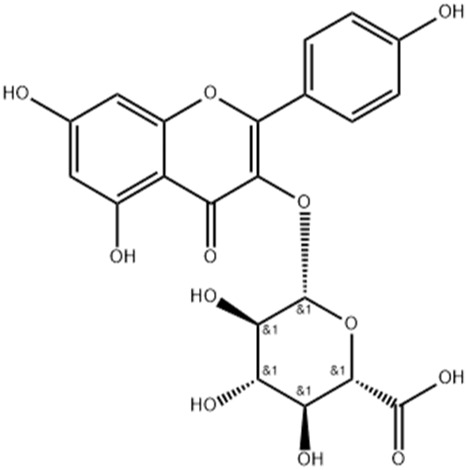
9.28	0.613	Kaempferol-3-rhamnoside-4″-rhamnoside-7-rhamnoside	C_33_H_40_O_18_	724.221	726.2865	2.0655	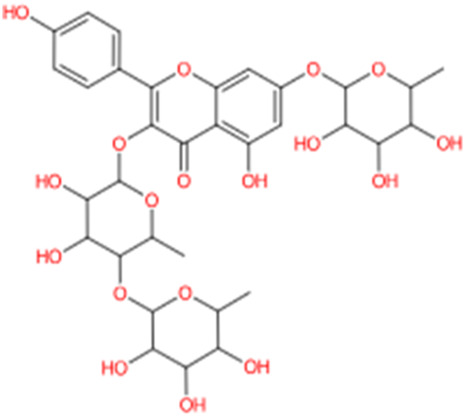
15.43	0.74	Cytidine-5′-diphosphocholine sodium salt dihydrate	C_14_H_26_N_4_O_11_P_2_	488.107	488.0018	0.1052	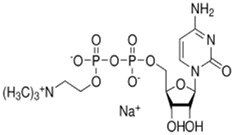
21.23	0.766	2′-deoxycytidine	C_9_H_13_N_3_O_4_	227.09	227.6646	0.5746	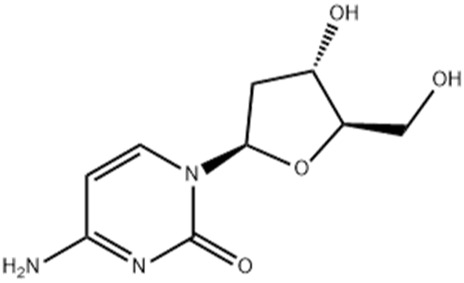
23.07	0.983	Gamma-linolenic acid	C_18_H_30_O_2_	278.43	279.6876	1.2576	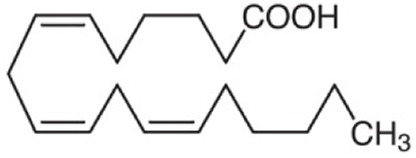
25.84	0.895	Daidzein	C_15_H_10_O_4_	254.25	255.6741	1.4241	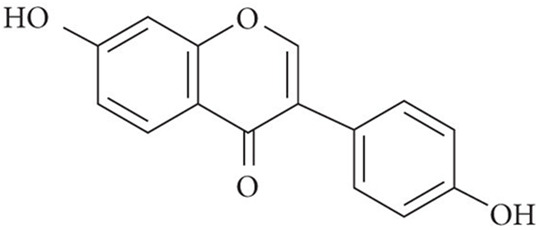
26.28	0.83	Xanthosine	C_10_H_12_N_4_O_6_	284.075	281.7226	2.3524	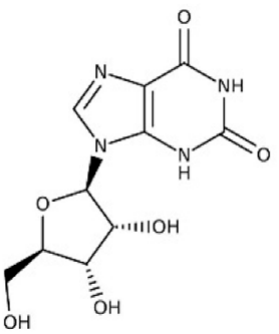
26.52	0.836	Acacetin	C_16_H_12_O_5_	284.068	281.7226	2.3454	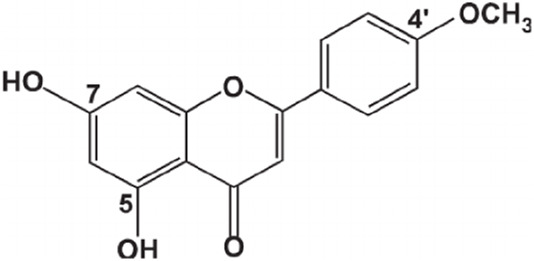
32.18	0.816	Luteolin	C_15_H_10_O_6_	286.047	283.7577	2.2893	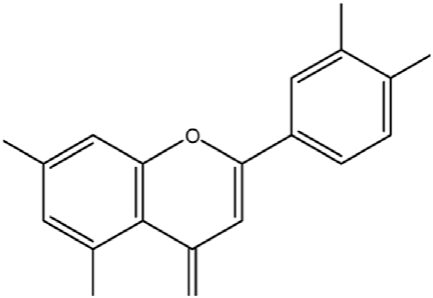

### 3.2 Antibacterial activity

**FIGURE 3:**
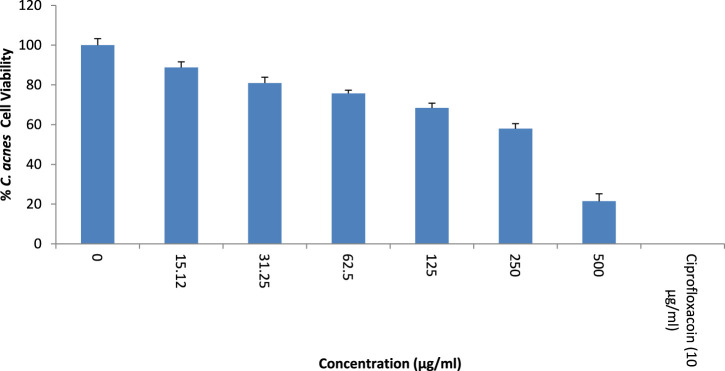
Antibacterial activity against *Cutibacterium acnes* at different concentrations of *T. oliverianum* extract, n = 4.

The growth inhibitory action of *T. oliverianum* extract is shown in Figure 3. There was a dose-dependent inhibition of *C. acnes* growth when the extract was tested up to a concentration of 500 μg/mL. The IC_50_ value of *T. oliverianum* extract against *C. acnes* was found to be 263.2 ± 0.061 μg/mL.

### 3.3 Cytotoxicity assay of *Teucrium oliverianum* extract on HaCaT cells by neutral red uptake assay

**FIGURE 4:**
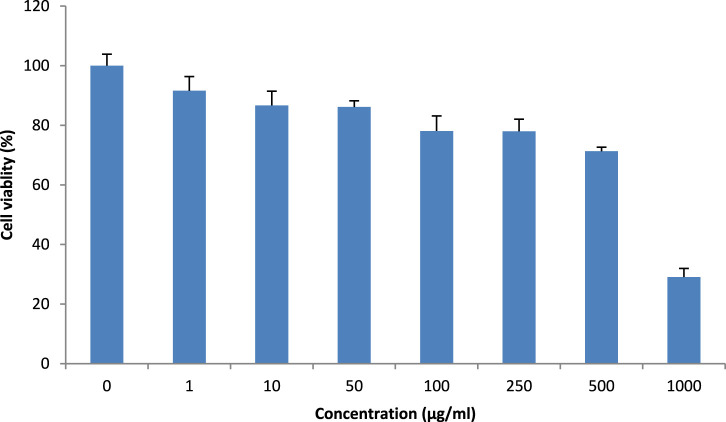
Cytotoxicity of *T. oliverianum* extract on HaCaT cells at varying concentration, n = 4.

The methanolic extract of *T. oliverianum* was safe on the HaCaT cell, as revealed by the cell viability when incubated with different concentrations (Figure 4). Cell viability of less than 80% was observed at concentrations above 250 μg/mL. The IC_50_ was found to be 676.2 ± 0.111 μg/mL, indicating its safety on HaCaT cells.

### 3.4 Anti-acne activity

**FIGURE 5:**
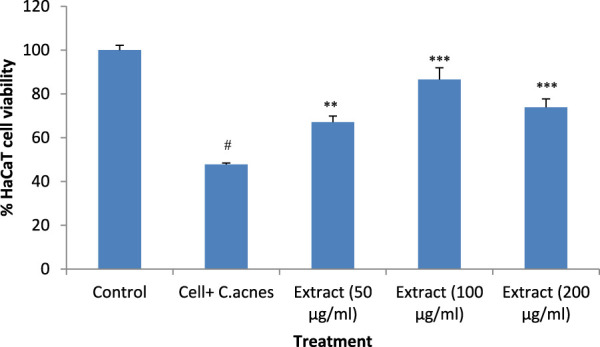
Cell viability after different treatments in HaCaT cells. Values are mean ± SEM, n = 4, ^#^ *P* < 0.001 as compared to negative control. **P < 0.01, ***P < 0.001 as compared to positive control.

Incubation of HaCaT cells with the microbe *C. acnes* reduced the cell viability (*P* < 0.001) compared to the control. The addition of the extract to the *C. acnes*-infected HaCaT cells prevented the cytotoxic effect of *C. acnes* significantly. The lower concentration of *T. oliverianum* extract (50 μg/mL) and medium concentration of the extract (100 μg/mL) showed more effect than 200 μg/mL, indicating that the maximum effective dose could be less than 200 μg/mL. The cell viability reduced by approximately 52% after infection with *C. acnes*. The addition of extract at 50 μg/mL increased the cell viability by around 20% when compared to *C. acnes* alone treated cells. The cell viability was maximum after the addition of 100 μg/ml *T. oliverianum* extract wherein the cell viability was around 86%, which was 39% more than *C. acnes* alone treated cells. However, a dose-dependent effect could not be observed as indicated by a non-significant decrease in cell viability in ml *T. oliverianum* extract (200 μg/mL) as compared to *T. oliverianum* extract (100 μg/mL (Figure 5)).

### 3.5 COX-2 enzyme inhibition assay

**FIGURE 6:**
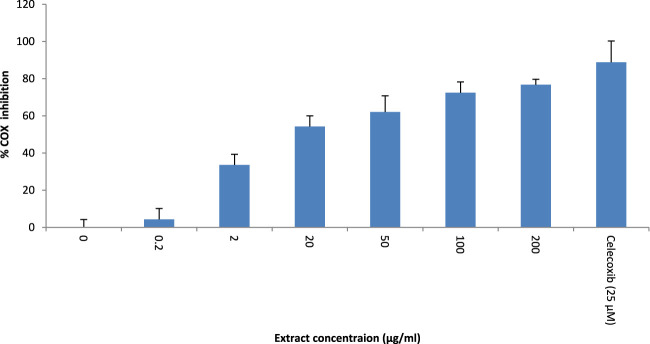
Enzyme inhibition assay-COX-2, n = 4.

*T. oliverianum* methanolic extract was effective in inhibiting COX-2 enzyme activity. The effect was dose-dependent up to 200 μg/mL, the maximum concentration tested. The IC_50_ was found to be 14.51 μg/mL. However, the effect was less than celecoxib, a selective COX-2 inhibitor (Figure 6).

### 3.6 TNF- α expression analysis

**FIGURE 7:**
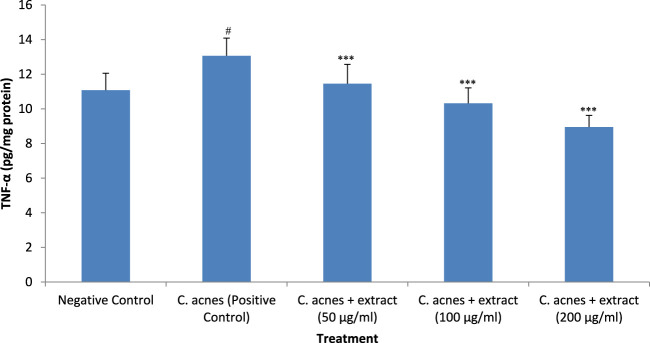
TNF-α levels after treatment with extract in HaCaT cells. Values are mean ± SEM, n = 4, ^#^ *P* < 0.05 as compared to negative control. ***P < 0.001 as compared to positive control.

Treatment of HaCaT cells with *C. acnes* increased the expression of TNF-α, a proinflammatory cytokine, significantly (*P* < 0.05) as compared to *C. acnes* HaCaT cells alone. The increase in the expression of cytokine after infection was around 18% as compared to control. The methanolic extract of *T. oliverianum* significantly inhibited the *C. acnes* induced expression of TNF-α dose-dependently when tested at 50, 100, and 200 μg/mL significantly (*P* < 0.001). The reduction in the expression of TNF-α by the *T. oliverianum* extract was 13%, 21%, and 32% with 50, 100, and 200 μg/mL of the extract respectively as compared to *C. acnes* infected cells (Figure 7).

### 3.7 IL-1β expression analysis

**FIGURE 8:**
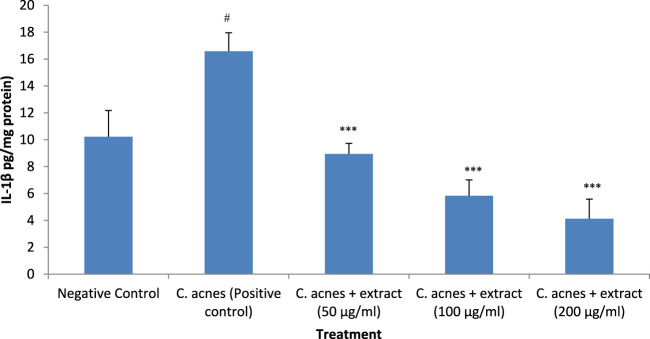
IL-1β levels after treatment with extract in HaCaT cells. Values are mean ± SEM, n = 4, ^#^ *P* < 0.05 as compared to negative control. ***P < 0.001 as compared to positive control.

The effect of *T. oliverianum* extract on the expression of IL-1β was similar to that observed with TNF-α expression. *C. acnes* increased the expression of this cytokine by 62%, which was significant when compared to the control (*P* < 0.05). The *T. oliverianum* extract at all the tested doses attenuated the expression of this cytokine (*P* < 0.001). The reduction in the expression was about 46% with the lowest concentration of the extract (50 μg/mL). The expression was reduced more with higher concentrations, wherein a decrease of 65% and 75% was seen at concentrations of 100 and 200 μg/mL respectively. No significant difference in the expression of IL-1β was observed between the treatment groups (Figure 8).

### 3.8 INF-γ expression analysis

**FIGURE 9:**
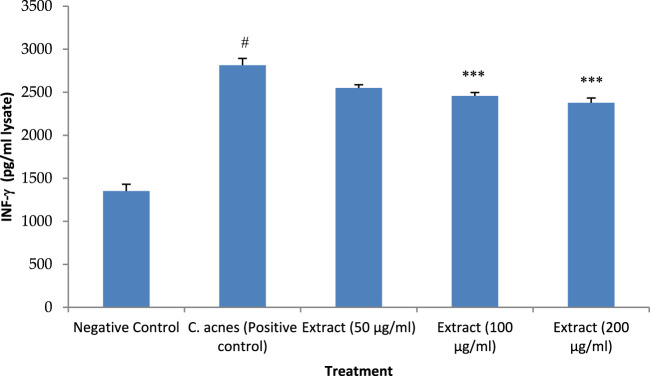
INF-γ levels after treatment with extract in HaCaT cells. Values are mean ± SEM, n = 4, ^#^ *P* < 0.001 as compared to negative control. ***P < 0.001 as compared to positive control.

Similar to the other cytokines, INF-γ expression was raised after infection of HaCaT cells with *C. acnes* by 108% which was significant as compared to the control (*P* < 0.05). The methanolic extract of *T. oliverianum* extract at low concentration (50 μg/mL) decreased the expression of INF-γ by 10%. However, this effect was not significant as compare dto control. But, the higher concentrations of 100 and 200 μg/mL significantly decreased the expression of INF-γ (*P* < 0.001) as compared to cells treated only with *C. acnes*. The reduction in the expression with 100 μg/mL was 13% while a reduction of 16% was seen with concentration of 200 μg/ml as compared to *C. acnes* treated cells (Figure 9).

### 3.9 ROS generation by flow cytometry

**FIGURE 10:**
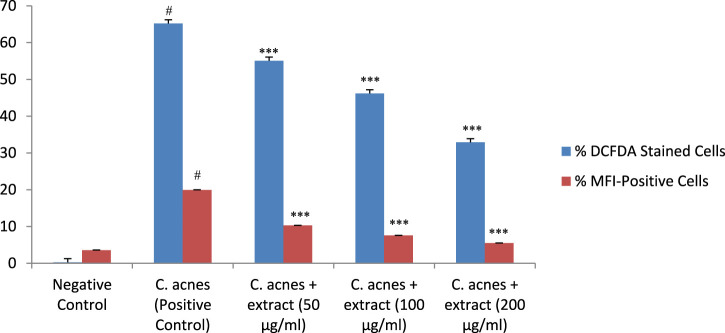
Percentage of DCFDA stained cells and percentage of MFI-positive cells indicating generation of ROS after treatment with extract in HaCaT cells. Values are mean ± SEM, n = 4, ^#^ *P* < 0.001 as compared to negative control. ***P < 0.001 as compared to positive control.

*C. acnes* infection of the HaCaT cells increased the generation of cytotoxic reactive oxygen species, as indicated by an increase in the percentage of DCFDA-stained cells (65%) and MFI-positive cells (19.9%) in *C. acnes* infected cells as compared to control (*P* < 0.001). *T. oliverianum* extract effectively prevented ROS generation in a dose-dependent manner as indicated by a reduction in the DCFDA-stained cells and MFI-positive cells as compared to *C. acnes.* There was no significant difference among the different concentrations (Figure 10).

## 4 Discussion

The results of the current study revealed the anti-acne activity of *T. oliverianum* due to its antibacterial effect against *C. acnes*, anti-inflammatory, and antioxidant effects. An analysis of the prepared methanolic extract of the aerial parts of the plant revealed the presence of some of the important metabolites, such as kaempferol, nicotine, and linoleic acid that have been reported for several pharmacological properties. The plant extract showed an antibacterial effect against *C. acnes*, the organism responsible for skin infections that lead to the formation of acne. Further evaluations of the anti-inflammatory actions showed it inhibits the COX-2 enzyme that is responsible for the formation of prostaglandins, an important mediator of inflammation. It also inhibited the expression of pro-inflammatory cytokines; TNFα, IL-1β, and INF-γ indicating multiple mechanisms for its anti-inflammatory effect. The extract also reduced the formation of ROS that causes cellular damage in the skin cells.

The current study was carried out using *C. acnes* to determine the antibacterial, antioxidant, and anti-inflammatory properties of *T. oliverianum* extract. *C. acnes* is part of normal flora in the skin, and it proliferates in the sebaceous glands by utilizing sebum. When there is an increase in sebum secretion in the skin, it causes an abundance of bacteria in the skin leading to the clogging of hair follicles, triggering an immune reaction that leads to inflammation and acne lesions (Komuro, 2017). The inflammation increases the expression of reactive oxygen species that further damages the skin (Chaudhary et al., 2023). Hence, agents with antibacterial, anti-inflammatory, and antioxidant effects are beneficial in treating acne.

The antibacterial study was carried out using conventional method and inhibitory concentration-50 (IC_50_) was taken as parameter to determine the antibacterial potential of the extract. The extract showed antimicrobial action at a high IC_50_ concentration of 263.2 μg/mL indicating a poor antibacterial effect. Different authors have proposed varying concentrations for an extract to be considered as a good antibacterial agent. Ríos and Recio (2005) suggest that for the antimicrobial activity of a plant extract to be considered significant, the MIC should be less than 100 μg/mL. Earlier studies on the antimicrobial effect of this extract against several bacteria showed a similar effect wherein it was not active against *Pseudomonas aeruginosa* and showed mild antibacterial action against *Staphylococcus aureus* and *Bacillus subtilis* (Fatima et al., 2017). In this study, the authors prepared four fractions from the alcoholic extract (70% ethanol) extract of the plant using n-hexane, chloroform, ethyl acetate, and n-butanol. All four fractions at a concentration of 20 mg/mL did not show any zone of inhibition against *P. aeruginosa*. Only the chloroform fraction showed a zone of inhibition of 6.6 mm against *S. aureus* which was less compared to standard antibiotic carbenicillin (2 μg/mL), which showed about 23 mm zone of inhibition. In our study, the MIC of the crude extract was high (263.2 μg/mL) and the results of our study support the earlier finding that the alcoholic extracts (methanol or ethanol) of *T. oliverianum* and their non-polar fractions have poor antibacterial activity. In the present study, DMSO (10%) was used to dissolve the extract based on the solubility to obtain a homogenous solution. There are earlier reports that DMSO affects the growth of some bacteria such as *P. aeruginosa*. However, earlier reports on the effect of DMSO on *C. acnes* show that DMSO up to 100% concentration does not inhibit the growth of this bacteria (Joo et al., 2022; Boonchaya et al., 2022).

Expression of three different cytokines was carried out. The TNF-α is known to promote inflammation by recruiting the immune cells to the inflammatory site leading to inflammatory cascade. It is also reported to promote apoptosis of infected cells and regulate the activity of T cells and B immune cells (Jang et al., 2021). Agents that inhibit TNF-α are therapeutically used into treat inflammation (Zhang et al., 2019). Similar to TNF-α, the IL-1β and IFN-γ are proinflammatory agents involved in inflammation and immune responses (Hommel et al., 2023; Gauthier and Chen, 2022). It has been shown that IL-1β mRNA levels, and the IL-1β levels are increased after *C. acnes* infection. Additionally, *C. acnes* triggers the activation of the NLRP3 inflammasome in monocyte-macrophage, leading to the release of IL-1β. Hence, targeting the NLRP3 inflammasome and IL-1β could be a potential therapeutic approach for acne (Kistowska et al., 2014). *C. acnes* infection can also induce the release of INF-γ and inhibiting INF-γ dependent feedback loop may inhibit acne and several other inflammatory diseases such as pyogenic arthritis, and pyoderma gangrenosum (Lee et al., 2024).

Several botanical drugs are traditionally used to manage acne, and many of these botanical drugs possess antibacterial or anti-inflammatory effects. *T. oliverianum* is commonly called Oliver’s germander and is native to Mediterranean countries, especially Spain and Morocco. It is cultivated as an ornamental plant in gardens and landscapes. The genus *Teucrium* contains almost 340 species and members of this family are reported for a wide range of biological effects (Fatima, 2016). LC-MS analysis of the extract revealed the presence of different classes of metabolites. There are no reports on the metabolites present in this plant. Hence, a comparison of the metabolites revealed in the current study could not be compared with earlier reports.

There are no reports on the anti-inflammatory and antioxidant activities of *T. oliverianum.* However, another plant from the same genus- *T. polium* has been reported for both anti-inflammatory and antioxidant effects. The anti-inflammatory effect of ethanolic extract of *T. polium* was reported *in-vivo* in rats using carrageenan-induced inflammation and cotton pellet granuloma models at doses ranging from 50 to 150 mg/kg in carrageenan-induced inflammation and at a dose of 500 mg/kg in cotton-pellet granuloma models (Tariq et al., 1989; Al-Naemi et al., 2024). The plant *T. oliverianum* belongs to the Lamiaceae family. A previous study on the ethanolic leaf extracts of five species from the Lamiaceae family demonstrated that they have anti-acne effects through tyrosinase inhibitory and antioxidant activity, suggesting potential for treating skin hyperpigmentation (Lambrechts et al., 2015). The minimum IC_50_ against *C. acnes* among all the above tested herbs was 7.8 μg/mL. Additionally, essential oils from four Elsholtzia species - *Elsholtzia stachyodes*, *Elsholtzia communis*, *Elsholtzia griffithii*, and *Elsholtzia beddomei* - belonging to the Lamiaceae family exhibited anti-acne activity by inhibiting the growth of *S. aureus* and *Staphylococcus epidermidis* (Phetsang et al., 2019). In this study, the most effective oil was from *E. stachyodes* that inhibited the growth of *S. aureus* and *S. epidermidis* with MIC values of 0.78 and 1.56 μL/mL, respectively. Furthermore, essential oils from lavender (*Lavandula angustifolia* Mill.), a shrub from the Lamiaceae family, are widely used for acne treatment due to their antibacterial and antifungal effects (Nurzyńska-Wierdak et al., 2023). The IC_50_ of lavender oil against *C. acnes* was shown to be 2.52 mg/mL (Taleb et al., 2018). Similarly, essential oils from oregano (*Origanum vulgare* L.) and thyme (*Thymus vulgaris* L.), both from the Lamiaceae family, are used for acne treatment because of their antimicrobial properties and it was shown to have an MIC of 0.65 mg/mL (Nurzyńska-Wierdak et al., 2023; Taleb et al., 2018). Basil (*Ocimum sanctum*), another popular herb from the Lamiaceae family, is used for acne treatment, and a water extract of this plant is reported to have a strong inhibitory effect against *C. acnes* (Zhang et al., 2022). The inhibition zone of water extract of basil against acnes was 18.71 mm in diameter, which was significantly higher than 10.87 mm observed with 0.1% salicylic acid, a standard drug used for comparison in the study (Zhang et al., 2022). Additionally, *Rosmarinus officinalis* from this family has been extensively studied for its anti-acne effects (Fernandes et al., 2023; Masoud et al., 2022). A rosemary gel applied to participants with acne reduced total number of inflammatory lesions (TIL), total number of comedones (TC), and pustules and papules (Masoud et al., 2022).

A literature review on the various predicted metabolites revealed that these metabolites have anti-inflammatory, antioxidant, and antibacterial properties. L-carnitine is a naturally occurring cofactor for the metabolism of fatty acids and is known for its antioxidant actions that include scavenging of free radicals, generation of antioxidant enzymes, and protection against lipid peroxidation (Di Emidio et al., 2020). Apart from this, L-carnitine is an immunomodulator and an effective anti-inflammatory agent (Rastgoo et al., 2023). The antioxidant, anti-inflammatory, and immunomodulatory actions may contribute to the anti-acne action. Hinokitiol is present in several species of plants and derives its name from Taiwan hinoki. It is reported for several biological effects, including antimicrobial, anti-inflammatory, and wound healing properties (Hachlafi et al., 2021). All these effects may contribute favorably to the anti-acne action.

Galactinol dehydrate is a carbohydrate used in skin care products to moisturize the skin. It is also reported to scavenge free radicals that may indirectly reduce the skin damage and inflammation associated with acne (Elango et al., 2022). Esculin sesquihydrate is abundantly found in many plants, especially chestnut trees. It has been reported to possess antioxidant, antimicrobial, and anti-inflammatory actions, all contributing to the anti-acne effect (Li et al., 2022). Furthermore, esculin sesquihydrate is an ingredient of cosmetic formulations used as anti-aging agents.

Kaempferol is an important flavonoid that is found in several plants and has been reported for diverse pharmacological actions (Periferakis et al., 2022). The anti-inflammatory effect of kaempferol has been studied in detail and is reported to reduce levels of cytokines both *in-vitro* and *in-vivo*. It is reported to decrease the levels of COX-2, TNF- α, interleukins, inducible nitric oxide synthase to name few mechanisms (Alam et al., 2020). Kaempferol is also a potent antibacterial agent that is reported to inhibit several species of bacteria (Periferakis et al., 2022). Local application of kaempferol is reported to increase wound healing through various mechanisms in both diabetic and non-diabetic animals (Özay et al., 2019). Kaempferol is also a potent antioxidant that is reported to scavenge several free radicals and decrease ROS generation (Diao et al., 2021). Linoleic acid is a fatty acid that has beneficial effects on the skin. It is reported to have anti-inflammatory and moisturizing properties on the skin (Kim et al., 2014). Acacetin is another metabolites with good antioxidant and anti-inflammatory effects. These actions, along with their effect on collagen support, help in skin care, and it is a constituent of several cosmetic products (Jeong et al., 2020). Similar to acacetin, luteolin has good antioxidant and anti-inflammatory effects. It is also used in skin care due to these properties along with its modulation of skin aging (Gendrisch et al., 2021).

The results of this study suggests that *T. oliverianum* has mild antibacterial effect along with good anti-inflammatory, and antioxidant effects. All these three effects might contribute to the antiacne effect of this extract. Further investigations on the isolated metabolites may provide more insight into the contribution of each metabolite to the observed effects.

The study has a few limitations. All the studies were conducted *in-vitro* using human keratinocytes, the HaCaT cells. The potential adverse effects that may be observed when applied to human skin and toxicological evaluation of the extract on the skin tissue should be carried out. Furthermore, the effectiveness of the extract may not be the same when applied to intact live skin. Hence, a direct extrapolation of the results of the current study to that observed clinically should be done only after clinical study reports are available.

## 5 Conclusion

The methanolic extract of *T. oliverianum* showed dose-dependent antibacterial, anti-inflammatory, and antioxidant effects on HaCaT cells *in-vitro*. LC-MS analysis of the extract revealed the presence of 23 different metabolites in positive and negative modes. *T. oliverianum* extract inhibited the growth of *C. acnes* and prevented the cytotoxicity of *C. acnes* on the HaCaT cell. The extract effectively prevented the expression of proinflammatory cytokines TNFα, IL-1β, and INF-γ from HaCaT cells. A decrease in the expression in ROS was also observed when HaCaT cells were incubated with *T. oliverianum* extract. The results support earlier findings on the mild antibacterial effect of *T. oliverianum* extract. The combined antibacterial, anti-inflammatory and antioxidant actions of the extract may help in reducing acne clinically.

## Data Availability

The raw data supporting the conclusions of this article will be made available by the authors, without undue reservation.
